# Genital Tract Sequestration of SIV following Acute Infection

**DOI:** 10.1371/journal.ppat.1001293

**Published:** 2011-02-17

**Authors:** James B. Whitney, Peter T. Hraber, Corinne Luedemann, Elena E. Giorgi, Marcus G. Daniels, Tanmoy Bhattacharya, Srinivas S. Rao, John R. Mascola, Gary J. Nabel, Bette T. Korber, Norman L. Letvin

**Affiliations:** 1 Division of Viral Pathogenesis, Department of Medicine, Beth Israel Deaconess Medical Center, Boston, Massachusetts, United States of America; 2 Harvard Medical School, Boston, Massachusetts, United States of America; 3 Los Alamos National Laboratory, Los Alamos, New Mexico, United States of America; 4 Vaccine Research Center, National Institutes of Allergy and Infectious Diseases, Bethesda, Maryland, United States of America; Fred Hutchinson Cancer Research Center, United States of America

## Abstract

We characterized the evolution of simian immunodeficiency virus (SIV) in the male genital tract by examining blood- and semen-associated virus from experimentally and sham vaccinated rhesus monkeys during primary infection. At the time of peak virus replication, SIV sequences were intermixed between the blood and semen supporting a scenario of high-level virus “spillover” into the male genital tract. However, at the time of virus set point, compartmentalization was apparent in 4 of 7 evaluated monkeys, likely as a consequence of restricted virus gene flow between anatomic compartments after the resolution of primary viremia. These findings suggest that SIV replication in the male genital tract evolves to compartmentalization after peak viremia resolves.

## Author Summary

Methods to reduce the transmission of HIV-1 are hindered by a lack of information regarding early viral dynamics and evolution in the male genital tract. In the present study, we show that SIV in the blood and genital tract are homogeneous during early infection, indicating facile virus gene flow between these compartments. Importantly, the coincidence of the resolution of primary viremia with the decreased virus levels in genital secretions suggest that the dramatic fall in virus replication during early infection underlies the development of viral compartmentalization. Our demonstration of early virus compartmentalization in the male genital tract has important implications for the understanding of early events leading to infection of the male genital tract and the nature of the transmitted virus during primary retrovirus infection.

## Introduction

An understanding of HIV-1 biology in the male genital tract will be central to understanding the transmission of this virus. Since HIV-1 is transmitted predominantly by sexual contact [1], [2], [3], semen represents an important source of transmitted virus. While the transmission risk of HIV-1 has been associated with virus RNA levels in the peripheral blood of infected transmitting individuals, virus RNA levels in the blood only serve as a surrogate for the level of virus in ejaculate [1], [3], [4], [5], [6]. Indeed, during primary infection when HIV-1 transmission is high, virus levels in both the blood and the seminal plasma are both elevated [1], [7]. Yet, during chronic infection blood and semen levels of HIV-1 can be discordant [1], [7].

HIV-1 exists as a diverse population of related genetic variants that segregate into subpopulations within anatomic compartments of infected individuals [8], [9]. The intra-host compartmentalization of these variants has been documented in multiple anatomic regions including the peripheral blood, lungs, central nervous system, breast milk, gut and the female genital tract, [1], [10], [11], [12], [13], [14], [15], [16].

Importantly, the male genital tract serves as a reservoir for HIV-1 that has been shown by some investigators to harbor virus that is distinct at the sequence level from virus found in the blood [12], [15], [17], [18], [19]. It has been suggested that this compartmentalization may be a consequence of differences between the male genital tract and peripheral tissues in both immunological and target cell properties [14], [15], [20], [21], [22], [23], [24], [25], [26], [27], [28]. Histopathologic studies of genital tissues from SIV-infected monkeys have confirmed that infection can occur at these sites [24], [29]. Male genital tract-derived viral variants have been reported to have unique phenotypes with regard to drug resistance [11], [30], [31], [32], [33] and cellular tropism [34], [35]. Some groups have reported that HIV-1 in the male genital tract [1], [36], [37], [38] is similar to that in the blood [39], while other groups have suggested that there is an anatomic sequestration of virus in some individuals during primary infection [10], [34], [35], [40], [41]. Overall, the mechanisms that contribute to compartmentalized virus in the male genital tract are not entirely clear. Virus compartmentalization could be the result of founder effects, differential immune selection, altered tropism or restricted gene flow between resident viral populations [42], [43].

In the present study we sought to define the dynamics of SIV compartmentalization in semen during primary infection in rhesus monkeys. We accomplished this by analyzing SIV envelope (*env*) sequences in the blood and semen at the peak and set point of SIV replication in rhesus monkeys. We observed a striking progression toward increasing viral compartmentalization over time following infection, raising the possibility that the abrupt reduction in virus replication that occurred in these monkeys after the resolution of peak viremia may contribute to a restriction in viral gene flow in and out of their genital tracts.

## Results

### Sequence analysis of contemporaneous blood- and semen-derived SIV during primary infection

Twenty-six rhesus monkeys were vaccinated and then monitored for 16 weeks after experimental SIV infection as previously described [44]. Thirteen were vaccinated using Gag-Pol immunogens with a heterologous prime/boost regimen that included both plasmid DNA and replication-defective recombinant adenovirus serotype 5 (Ad5) immunogens. The remaining 13 control monkeys received sham vaccine constructs. Animals were challenged by the i.v. route using a SIVmac251 stock with a previously reported sequence diversity [45].

To determine whether virus in the semen and peripheral blood were compartmentalized following infection, we assessed the phylogenetic relationship between semen- and blood-derived SIV *env* sequences. Single genome amplification (SGA) was used to generate 789 *env* amplicons for sequencing and subsequent phylogenetic analysis. Sequence data were generated from 2 time points post-challenge, at week 2 (peak virus replication) and week 16 (virus set point).

Viral RNA levels were comparable for each group, and we detected no significant differences in week-2 and week-16 blood or seminal plasma viral loads between vaccinees and controls. Similarly, no significant differences were detected between animals included in this study and animals excluded as a result of insufficient sequence data (Wilcoxon P>0.01) as described [44].

**Figure 1:**
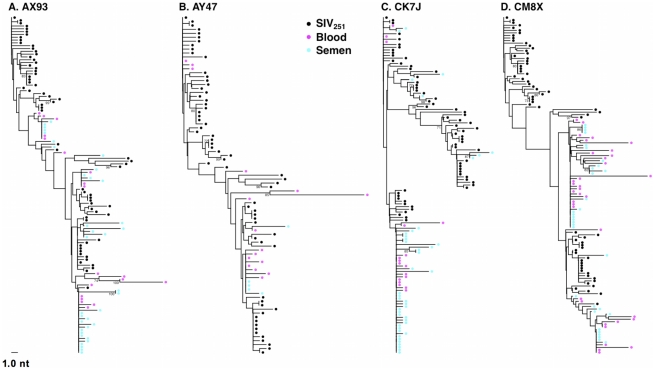
Intermixing of blood- and semen-derived *env* sequences during peak SIV replication. Neighbor-joining trees from vaccinated monkeys (**A**) AX93, (**B**) AY47 and control monkeys (**C**) CK7J and (**D**) CM8X 2 weeks after challenge with a sequence-defined SIVmac251 challenge stock (stock sequences denoted in black).

SIV *env* sequences were first generated from both the blood and semen of 14 rhesus monkeys (7 vaccinated and 7 control) from specimens obtained 2 weeks following infection. The genetic diversity of the virus from each anatomic compartment of each monkey was calculated as the average pair-wise genetic distance of SIV *env* within each semen or blood population from the inoculum consensus. Data from the vaccinated and control animals were analyzed independently. Since these represented analyses of sequence from virus obtained very early after infection, little divergence was seen from the founder viruses that initiated infection in each monkey (**Table 1**). Moreover, we observed little genetic diversity between sequences of blood and semen amplicons at this early time point after challenge, representative examples from 4 monkeys (2 control, 2 vaccinated) are shown (**Fig. 1**). Among the sequences from the blood, we observed no difference in average pairwise *env* distance from the inoculum consensus per animal between vaccinees (0.29% versus 0.28% respectively, P = 0.9). The sequence divergence for all week 2 semen isolates were not significantly different in control and vaccinated monkeys, with a mean distance among control animals of 0.27% and 0.15% among vaccinated monkeys (P = 0.4). These differences are consistent with the lower levels of viral replication in the vaccinated group [44].

**Table 1:** Quantity and diversity of sequences obtained per sample.

ID	Vaccinee/Control	Time (Weeks Post-infection)	Blood Seqs.	Semen Seqs.	Informative Sites	MPD (%)
SIVmac251 inoculum	NA	NA	61	23	0.373	
AX89	C	2	27	52	43	0.459
		16	29	3	41	0.683
C161	C	2	31	4	17	0.351
C171	C	2	21	2	16	0.230
		16	17	24	19	0.308
CK7J	C	2	17	43	26	0.178
CM8X	C	2	35	32	21	0.298
CP6E	C	2	9	12	12	0.288
DA2D	C	2	14	2	23	0.465
		16	32	4	11	0.348
AX93	V	2	19	36	30	0.322
		16	39	9	36	0.484
AY11	V	2	22	3	16	0.310
		16	13	3	10	0.302
AY28	V	2	11	3	3	0.083
AY47	V	2	16	5	20	0.342
AY89	V	2	4	12	12	0.253
		16	38	27	32	0.407
C179	V	2	9	35	19	0.289
		16	19	25	21	0.313
D135	V	2	12	4	10	0.168

SIV *env* clones from control (**C**) and vaccinated (**V**) monkeys were amplified by SGA from semen and blood plasma 2 or 16 weeks after infection with a sequence-defined SIVmac251 stock. Shown are the number of sequence clones in blood and semen, the number of phylogenetically informative sites, and the Mean Pairwise Distance from the challenge stock.

We next analyzed blood and semen sequences from specimens obtained at week 16 following infection. Post set point, we were able to obtain sufficient numbers of concurrent semen- and blood-derived SIV sequences from 7 monkeys to allow a phylogenetic analysis of these viruses. Consistent with the lower level of virus replication in the vaccinated group, we observed reduced diversification of sequences obtained from the vaccinated monkeys, particularly in the seminal plasma. Specifically, blood-derived *env* sequences from control animals showed a trend towards greater divergence (0.45%) than *env* sequences derived from the blood plasma of vaccinees (0.35%, P = 0.2). In week 16 sequences obtained from seminal plasma, the mean pair wise distance per animal was 0.31% and 0.22% for controls and vaccinees, respectively (P = 0.2). We next analyzed these sequence data using available algorithms to determine if changes in *N-*linked Env glycosylation sites had occurred during the sampling period [46]. No significant changes in potential Env glycosylation sites were observed.

We then analyzed the sequences from the inoculum and week 16 samples for evidence of selection on SIV *env* by fixed-effects likelihood (FEL) [47]. As the results of this test can be influenced by the presence of recombinant sequences, we first used G.A.R.D. [48] to test for recombination and, if significant breakpoints (p<0.01) resulted, performed FEL tests separately for sequence alignments partitioned at the breakpoints.

The presence of negative selection could not be determined as intra-host viral populations had not diversified sufficiently for such events to occur at detectable levels. The positive selection tests that reached significance (p<0.05) are indicated (**Table S1**). Among the sequences from inoculum and week 16 sequences, we found 12 codons in 6 monkeys (3 vaccinees and 3 from the control group) under positive selection. One of these codons was under positive selection in 2 different animals (codon 427 in AX93 and AY47).

A number of investigators have postulated that specific amino acid signatures may be associated with HIV found in the semen [17], [49], [50]. To investigate this possibility, we applied a tree-corrected contingency table method to identify compartment specific amino acid signatures [51]. This analysis yielded no evidence for specific signatures that might be associated with SIV tropism in the semen up to 16 weeks following infection.

We then conducted additional signature analyses using only compartmentalized animals, but a sample size of 4 compartmentalized animals would lack sufficient statistical power to identify robust predictive signatures of compartmentalization. Therefore, we adapted a related strategy used by Pillai et al. [49]. Pillai reported a combination of HIV ENV residues (HXB2 270, 291, 387, and 464) associated with blood-semen compartmentalization. All of these except the first are located 1-2 residues downstream of putative N-linked glycosylation sites. We evaluated these sites in the SIV *env* sequences generated in this study by identifying homologous sites in HIV-1 and SIV from an alignment that incorporates HIV-1, HIV-2, HXB2, and SMM239 Seqs. (http://www.hiv.lanl.gov/content/sequence/NEWALIGN/align.html).

Reviewing the homologous signature sites in the SIV env sequences, we found 3 of 4 to be located 1–2 residues downstream of glycosylation sites. However, mutations in these sites (and in the neighboring 4 residues) were equally common in sequences derived from blood and semen, regardless of whether we included only sequences from compartmentalized animals. Thus, we were unable to confirm semen signatures in these SIV data.

### Unrestricted viral gene flow between the blood and genital tract during early SIV infection

We then sought to determine if SIV compartmentalization was demonstrable during primary infection. To detect compartmentalization in contemporaneous blood- and semen-derived SIV *env* sequences, we applied both phylogenetic analyses and the Slatkin-Maddison (SM) test from the HYPHY software package (v0.99b) [52], [53]. At the peak of SIV viremia, similar *env* sequences were observed in the control and vaccinated animals. Importantly, we observed the mixing of *env* taxa from blood- and seminal plasma-derived virus, and the association of these sequences into closely related phylograms (**Fig. 1**). Analysis of the week 2 blood and seminal virus sequences indicated that all 14 monkeys are similar on the basis of Neighbor-Joining (NJ) tree topologies. Importantly, we observed clusters of intermixed monotypic blood and semen sequences with minimal genetic divergence from the challenge SIV *env* sequences, termed “clusters of identity” in all animals, although the amount varied between individual monkeys (**Fig. 1**).

Representative NJ phylogenetic trees generated from SIV sequences from control monkeys CK7J and CM8X (**Figs. 1 A, B**), and from vaccinated monkeys AY47 and AX93 (**Figs. 1 C, D**) are shown. In control monkey CK7J, we observed one cluster of closely related semen and blood-borne sequences in the phylogenetic tree. In control monkey CM8X, two clusters of intermixed blood/semen sequences are apparent in the tree structure. Similar results were observed in the Gag-Pol vaccinated monkeys. For example, in vaccinated monkey AY47 a single phylogenetic cluster of interspersed semen and blood *env* sequences are apparent in the tree. Monkey AX93, has three clusters of identical, monotypic semen and blood sequences (**Fig. 1 C and D**).

**Figure 2:**
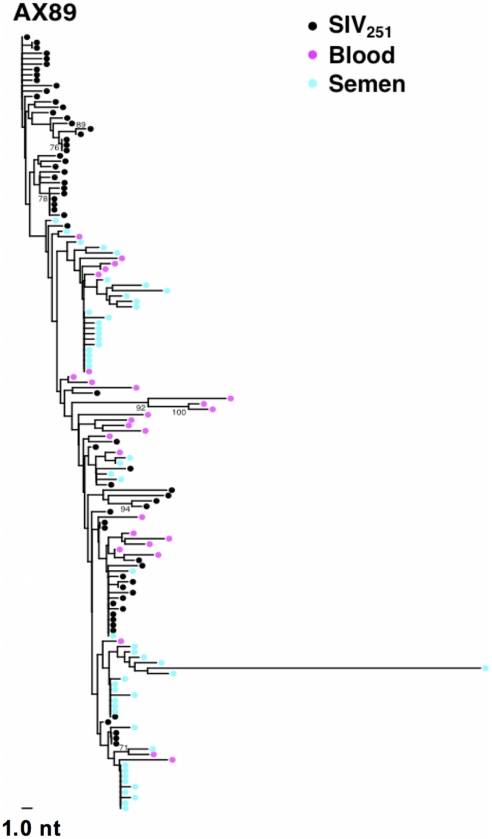
Compartmentalization of blood- and semen-derived *env* sequences during peak SIV replication in monkey AX89. NJ tree from control animal AX89, 2 weeks after challenge using a sequence-defined SIVmac251 stock (denoted in black).

While we did not observe topological evidence for virus compartmentalization between the semen and blood during early infection in any of the 14 monkeys analyzed, compartmentalization effects were also assessed using the SM test. Applying this metric, we observed no evidence of virus sequestration in week 2 blood- or semen-derived sequences in 13 of the 14 monkeys (Table 2). Control monkey AX89 had compartmentalized semen *env* sequences (s = 14, P<0.0001) (Fig. 2). However, the presence of large numbers of identical monotypic sequences might bias statistical measures of compartmentalization [54], [55]. To ensure that the large numbers of isogenic sequences in the semen of AX89 were not confounding this statistical analysis, we conducted a secondary analysis that collapsed all equivalently rooted monotypic sequences to a single representative *env* taxon. We then applied the same SM test to the normalized set of *env* sequences from monkey AX89. This modified analysis also indicated significant compartmentalization between SIV *env* in the blood and the male genital tract of monkey AX89 (s = 14, P = 0.0007). Therefore, there was weak evidence for virus compartmentalization in only 1 of the 14 monkeys by 2 weeks post-infection.

**Table 2:** SIV genetic compartmentalization at peak virus replication.

ID	Vaccinee/Control	wk 2 blood n =	wk 2 semen n =	s	N(s'≤s)	P
AX89	C	27	52	12	0	<0.0001***
C161	C	31	4	3	950	0.095
C171	C	21	3	2	10000	1
DA2D	C	14	3	2	10000	1
CK7J	C	17	43	13	1325	0.1325
CM8X	C	35	32	22	4873	0.4873
CP6E	C	9	12	5	932	0.0932
D135	V	12	4	3	2464	0.2464
AX93	V	19	36	14	148	0.148
AY11	V	22	3	3	10000	1
AY28	V	11	3	2	10000	1
AY47	V	16	5	5	10000	1
AY89	V	4	12	4	10000	1
C179	V	9	35	8	4286	0.4286

SIV *env* clones from control (**C**) and vaccinated (**V**) monkeys were amplified by SGA from semen and blood plasma 2 weeks after infection with a sequence-defined SIVmac251 stock. Shown are the number of clones per compartment (**n**), the number of Slatkin-Maddison migration events (**s**), and the number of permuted replicates having at most **s** migrations (**N(s'≤s)**) with associated P values (**P**) from 10,000 replicate permutations.

### Origins of virus in the male genital tract during early infection

**Figure 3:**
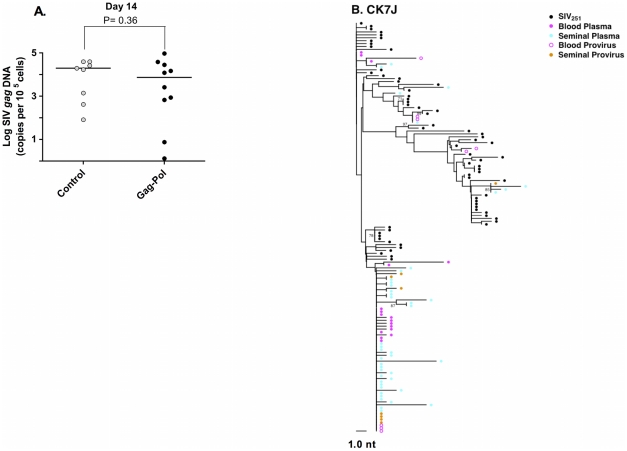
Source of SIV in the semen-associated cell fraction. (**A**) Semen virus DNA levels in the control and Gag-Pol vaccinated monkeys 14 days after virus challenge. The comparisons of the data from the Gag-Pol vaccinated and control groups were analyzed using the Mann-Whitney non-parametric T-test. The absolute number of SIV *gag* DNA copies was calculated as described previously [44], and is shown as log-transformed copies per 1×10^5^ semen-associated cells. (**B**) Phylogeny of SIV *env* in monkey CK7J, 14 days after virus challenge with blood plasma virus, semen plasma and semen cell-associated virus indicated.

We next explored the source of virus found in the male genital tract at the time of peak viremia. By day 14 following infection, significant levels of cell-free virus RNA and cell-associated provirus are present in the semen of both control and vaccinated animals (**Fig. 3A** **)** [44]. Examination of SIV *env* sequences from seminal plasma and semen-associated cells from monkey CK7J using NJ- tree parameters (**Fig. 3B**) indicated that several clusters of monotypic cell-free virus were identical in sequence to the semen provirus. Moreover, several clusters of cell-free and cell-associated virus in the genital tract of this monkey were phylogenetically indistinguishable from SIV *env* sequences derived cell-free and cell-associated virus from the blood. These data argue that in early SIV infection, during the period of peak viremia, there is significant virus gene flow between anatomic compartments. There are also significant levels of cell-associated and cell-free sources of virus present early in infection that may constitute a source of transmissible virus.

### Male genital tract SIV compartmentalization is observed at virus set point

**Figure 4:**
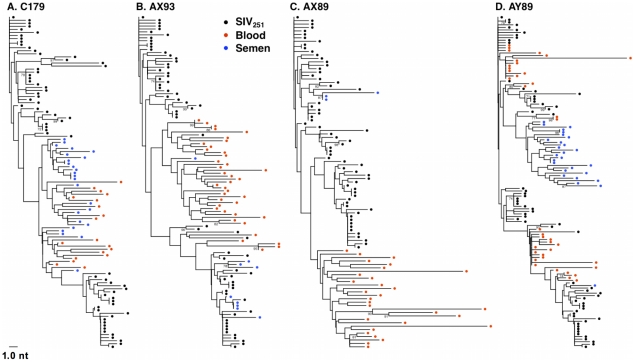
Anatomic sequestration, and diversification of SIV during set point virus replication. By 16 weeks after SIV challenge, compartmentalization of virus in semen and blood was apparent in 4 of 7 animals on the basis of tree topology and the SM test. NJ trees of SGA-derived *env* amplicons for compartmentalized monkeys (**A**) C179, (**B**) AX93 (**C**) AX89, and (**D**) AY89 are shown. Note that monkey AX89 is a control monkey and C179, AX93 and AY89 are vaccinated animals.

By week 16 following infection, we could readily detect virus compartmentalization by phylogenetic inference in 4 of the 7 monkeys that had sufficient numbers of sequences for analysis (Fig. 4). Compartmentalization is evident in these trees as independent clades consisting of sequences from one a single sample source, i.e. blood or semen. Included as a Supplement, are trees that combine sequencing data from both compartments at both acute and chronic time points (Fig. S1–S10 in Text S1). Typically, we observed that week 16 semen sequences tend to not be associated with those from week 2, rather they are evident as independent clades.

We also conducted an analysis, as described above, to ensure that isogenic sequences were not confounding the statistical analyses. This modified analysis did not significantly alter the findings as determined by tree topology. We also applied the SM test to phylogenies obtained from excluding duplicate sequences (Table 3) and found that, after correction for multiple testing, two animals, C179 and AX89, had marginally significant SM values (denoted P_n_).

**Table 3:** SIV genetic compartmentalization at virus set point.

ID	Vaccinee/Control	wk 16 blood n =	wk 16 semen n =	s	N(s'≤s)	P	P_n_
C171	C	17	24	12	2963	0.2963	0.7035
DA2D	C	32	4	4	10000	1	1
AX89	C	29	3	1	9	0.0009***	0.0250*
C179	V	19	25	10	118	0.0118*	0.0233*
AX93	V	39	9	2	0	<0.0001***	<0.0001***
AY11	V	14	3	2	10000	1	NA
AY89	V	38	27	1	0	<0.0001***	<0.0001***

SIV *env* clones from control (**C**) and vaccinated (**V**) monkeys were amplified by SGA from semen and blood plasma 16 weeks after infection with a sequence-defined SIVmac251 stock. Shown are the number of clones per compartment (**n**), the number of Slatkin-Maddison migration events (**s**), and the number of permuted replicates having at most **s** migrations (**N**(**s'≤s**)) with associated P values from 10,000 replicate permutations, and P values from trees normalized by eliminating multiple copies of identical sequences (**P_n_**).

**Figure 5:**
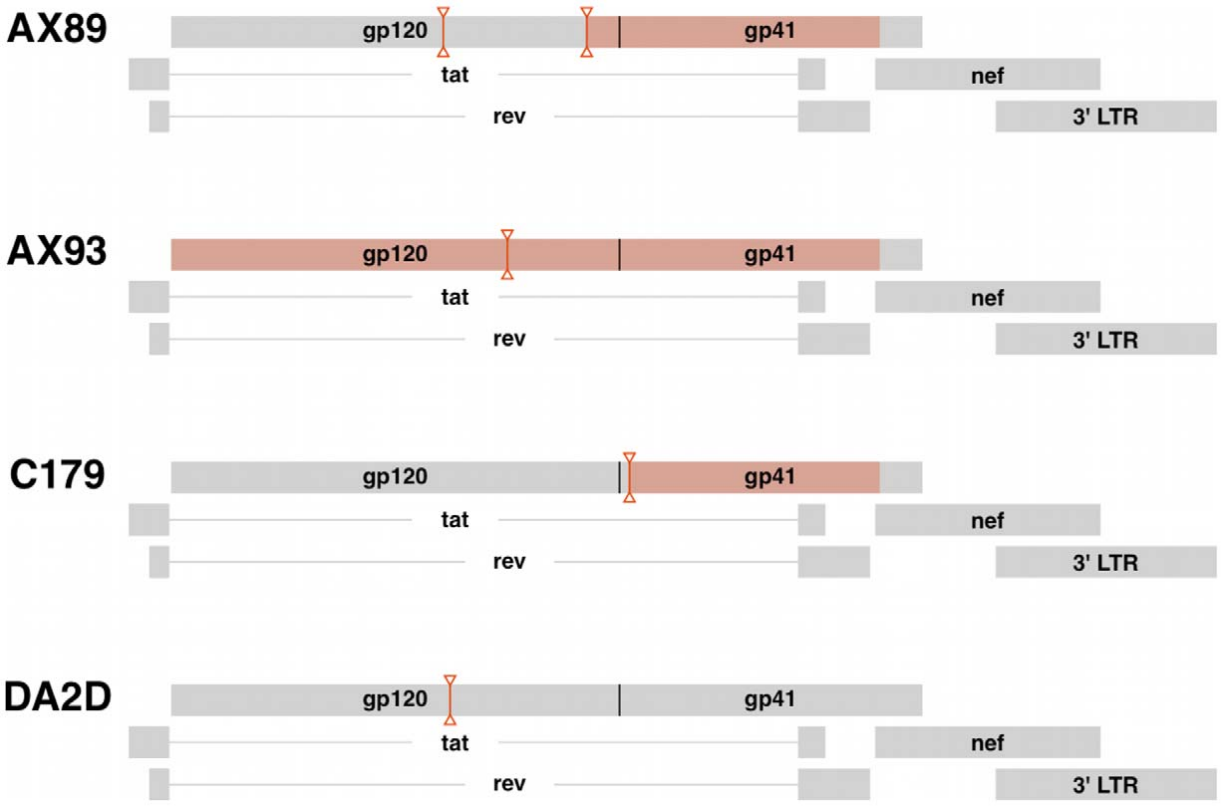
Virus recombination obscures compartmentalization of SIV *env* sequences. Diagrams indicating the positions of predicted recombination breakpoints in compartmentalized monkeys AX89, AX93 and C179 and non-compartmentalized monkey, DA2D. Recombination effects were determined by G.A.R.D. analysis as described. Breakpoint partitions are indicated as vertical lines between inverted triangles. SIV *env* regions with significant compartmentalization as determined by the partitioned SM test on partitioned sequences, **P<0.0002** are highlighted in red boxes.

Our inability to demonstrate definitive compartmentalization of virus using these strategies led us to consider whether other biologic events might be obscuring compartmentalization. Therefore, we evaluated the possibility that recombination may confound the SM analysis. To assess recombination effects we applied the G.A.R.D. algorithm [48], [56] and found significant levels of recombination (P<0.001) within SIV *env*, (data not shown). Next, we analyzed our data using a “segmented” SM-test based on *env* recombination breakpoints (not shown) and found specific regions of the SIV envelope from RMs C179 and AX89 were in fact significantly compartmentalized (P<0.0002, **Fig. 5**).

### Virus migration between compartments

**Figure 6:**
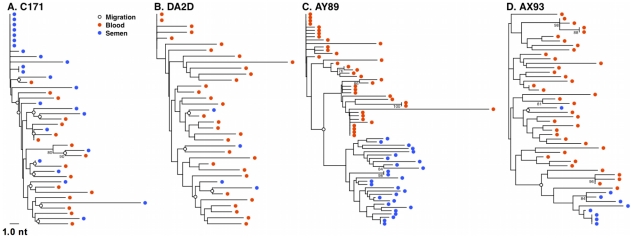
Restricted gene flow between the blood and semen is temporally associated with compartmentalization. SM migration events were plotted directly onto NJ trees for the 4 monkeys characterized 16 weeks after SIV challenge. Unrestricted viral gene flow between the blood and semen in monkeys C171 (**A**) and DA2D (**B**), and restricted gene flow in compartmentalized monkeys AY89 (**C**) and AX93 (**D**).

We next determined the relative levels of virus migration between the blood and semen by computing the minimum number of SM migration events for each inferred phylogenetic tree. These migrations occur where an internal node exhibits a change of sample source (blood or semen) among immediate descendents of that node. When these migration points were mapped directly onto phylogenies, we observed a concordance of semen compartmentalization with the number of migration events between these anatomic compartments, in individual monkeys. For example, the frequent migration events between the blood and male genital tract, as shown for monkeys C171, and DA2D (**Fig. 6A and B**), are consistent with unimpeded gene flow resulting in homogenous co-evolving SIV *env* populations. Restricted migration, as shown for monkey AY89 and AX93 (**Fig. 6C and D**) is consistent with the independent evolution of virus replicating in the blood and male genital tract. Therefore, we have documented examples of both unimpeded and restricted gene flow with the latter manifesting itself as virus compartmentalization.

### Mechanism of SIV compartmentalization in the male genital tract

We have therefore shown there was no evidence of SIV compartmentalization between the blood and semen in 13 of 14 monkeys analyzed at the peak of virus replication during primary infection. However, SIV compartmentalization was evident in 4 of the 7 analyzed monkeys by the time of virus set point.

**Figure 7:**
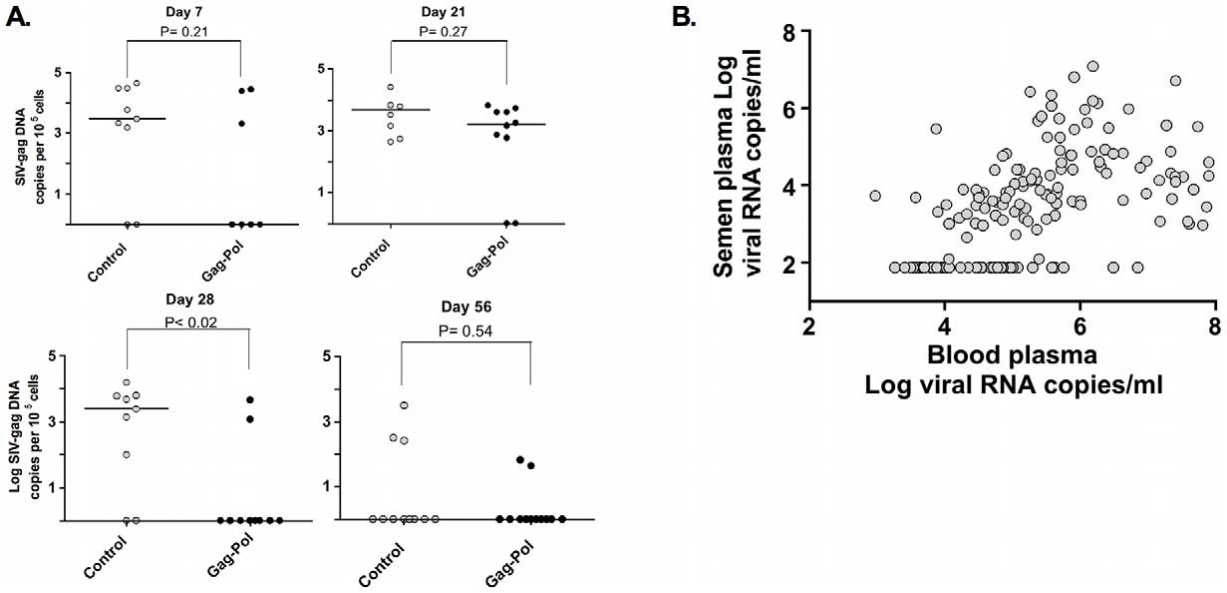
Association and threshold-effect between SIV DNA, and RNA levels in blood and semen. Cell-associated SIV trafficking into the male genital tract during peak and set point SIV infection. The comparisons of the data from the Gag-Pol vaccinated and control groups were analyzed using the Mann-Whitney non-parametric U-test. The absolute number of SIV *gag* DNA copies was calculated as described previously, and is shown as log-transformed copies per 1×10^5^ semen-associated cells. (**A**). Association and threshold effect between levels in blood plasma and seminal plasma from both vaccinated and control monkeys (**B**). Adapted from Whitney et al. [44].

To explore the mechanisms responsible for this changing pattern of viral evolution in the male genital tract, we evaluated 2 virologic correlates associated with compartmentalization. First, we observed that cell-associated virus levels in the male genital tract during peak infection are diminished significantly in the animals by week 8 following infection (**Fig. 7A**). Second, cell-free virus levels in the semen are also significantly diminished between weeks 2 and 16 following infection (**Fig. 7B**). In fact, these cell-free virus data demonstrate a threshold effect whereby viral RNA is only detectable in the seminal plasma when virus levels in the blood plasma exceed 10^4^ RNA copies/ml. The coincidence of the resolution of primary viremia with the decrease in levels of virus in genital secretions suggest that the dramatic fall in virus replication, with the accompanying restriction of gene flow, during this early period of infection underlies the development of viral compartmentalization.

## Discussion

While HIV-1 transmission in humans occurs most frequently through mucosal exposure, the present study was undertaken using rhesus monkeys infected by intravenous inoculation of SIV using artificial means to collect longitudinal semen samples. Although infection by this virus and route therefore does not model mucosal HIV-1 acquisition, the present findings have important ramifications for the understanding of early events leading to infection of the male genital tract, and semen as a source of transmitted virus. Low-risk sexual transmission of HIV-1 usually results in an infection initiated by a very limited number of founder viruses [45], [57], [58]. These viruses very rapidly appear in the blood after the transmission event, presumably as a consequence of the larger number of target cells in the blood than in the genital tract. In the periphery, HIV or SIV rapidly replicates to high levels and any subsequent virus diversification is likely a consequence of differences in virus replication in these compartments, independent of how infection was initiated.

During peak SIV replication we found little evidence for genetic segregation of virus between the semen and blood of infected rhesus monkeys. Instead, we observed an equilibration of SIV between these compartments manifested as closely related clusters of intermixed virus sequences derived from the semen or blood. As we applied SGA technology to generate all sequences [59], [60], the large numbers of identical sequences observed at peak virus replication are not likely to be a methodologic artifact. Moreover, since the SIV population size in the semen during peak virus replication was high, error as a result of re-sampling seems unlikely [61].

Based on the phylogenetic relationship among virus in the semen at peak replication, significant amounts of virus appear to be derived as a result of clonal expansion of infected cells. The homeostatic proliferation of SIV infected cells trafficking from the blood into genital tissues is a plausible means of producing the oligoclonal “bursts” of virus observed in ejaculate. These findings account for the magnitude and delay in peak semen viremia relative to the blood that has been reported in acutely HIV-1 infected patients [7], [36], [44]. Moreover, the influx of large numbers of SIV-infected cells into genital tissues make it unlikely that a specific virus amino acid “signature” could be a determinant of early viral tropism in the male genital tract [17], consistent with our inability to identify SIV Env amino acids that were significantly enriched in the seminal plasma. However, our analysis did not span a sufficient period of time to determine the presence of semen signatures during chronic infection [49], [50].

Mechanistically, the high-level of virus present in the blood during early infection results in a “wave” or spillover of cell-free and cell-associated virus into the male genital tract, equilibrating virus between the two compartments. Host control of SIV replication results in a receding “tide” of virus as virologic set point is achieved. When peak virus replication is reduced to levels below the threshold required for intra-compartment gene flow, free virus or infected cells may be effectively “trapped” within the genital tissues at “low tide.” Continued replication of virus in the male genital tract, in the absence (or reduced rate) of inter-compartment gene flow would then account for the localized diversification of virus variants in the genital tissues.

In fact, we observed less *env* diversification in the genital tract than in the blood of these monkeys. This finding is consistent with the lower level of SIV replication in the genital tract than in the blood at 16 weeks following infection. Moreover, the difference in virus diversification in these two anatomic compartments was particularly evident in the vaccinated monkeys after challenge (i.e., 3 of the 4 compartmentalized monkeys), consistent with the low-level virus replication in the male genital tract after set point [44]. Vaccine-elicited immune responses and alterations in target cells in the genital tract may have also contributed to the observed effects.

Although the male genital tract is generally considered to be an immune privileged compartment, inflammatory conditions present during acute SIV infection may facilitate the infiltration of both free virus and infected cells. Over time, differences between the blood and the genital tract, as a result of restrictions in gene flow would foster local replication in the genital tract leading to the compartmentalization observed at set point. Inflammation is a likely cause for the trafficking of cells from the peripheral circulation into the genital compartment, and may explain the observations of intermittent viral shedding in the semen of chronically HIV-infected men as a result of bacterial or viral infection of the male genital tract [34], [62], [63], [64], [65]. We did not determine if genital tract infections were present in any of the animals in this study.

These apparent differences in selective pressure between the blood and male genital tract should also be considered in light of the reduced effective SIV population size in the genital tract as virus set point is achieved. Virus populations of reduced size and altered genotype relative to the blood may contribute to the described genetic bottleneck associated with HIV-1 transmission, highlighted by several investigators [58], [66], [67]. This bottleneck is manifested by the acquisition of HIV-1 variants in newly infected individuals that are not highly represented in the peripheral blood of the already infected, transmitting partner. The findings in the present study, despite limitations in experimental infection route and virus dose, suggest an alternative explanation for these observations. Thus, the early compartmentalization of virus in the male genital tract, vis-à-vis restrictions in virus gene flow, may account for the transmission of distinct viral quasispecies that are not represented in the blood of the already-infected individual at the time of transmission.

## Materials and Methods

### Ethics statement

All Indian-origin rhesus monkeys used in this study and analysis were maintained according to the guidelines of the NIH Guide to the Care and Use of Laboratory Animals and the approval of the Institutional Animal Care and Use Committee of Harvard Medical School and the National Institute of Health. All monkeys were housed in a facility fully accredited by the Association for Assessment and Accreditation of Laboratory Animal Care International (AAALAC). All procedures were conducted in strict accordance with the Guide for Care and Use of Laboratory Animals, and approved by the Institutional Animal Care and Use Committee (IACUC) of Harvard University.

### Animals, vaccinations and SIV infection

This study monitored 26 adult Mamu-A*01 negative male rhesus monkeys (*Macaca mullata*) that were 4–6 years of age. All animals were confirmed as Mamu-A*01 negative by both PCR and MHC-amplicon sequencing. All animals were screened and found to be negative for simian immunodeficiency virus (SIV) and simian retrovirus type D (SRV-1) prior to the initiation of this study. All experiments were conducted in accordance with IACUC standards. Prior to, and routinely after SIV infection, all animals were monitored by physical exam, CBC with differential, and T cell subset analysis. The monkeys were housed under Biosafety level-2+ conditions.

Rhesus monkeys were immunized using a heterologous prime/boost regimen that included both plasmid DNA and replication-defective recombinant adenovirus serotype 5 immunogens. One group of 13 monkeys received these vaccine vectors expressing SIVmac239 *gag*, and *pol*, and a second group of 13 monkeys received sham vaccine constructs. The monkeys were challenged by the intravenous route with a pathogenic SIVmac251 quasispecies, 16 weeks after the last immunization. The experimental challenge consisted of injection with a 1 ml bolus of SIVmac251 (2.11×10^5^ viral RNA copies), directly into the saphenous vein.

### Sample collection and processing

Viral levels were monitored in the semen and blood for 16 weeks after infection. Specimens were collected weekly for the first 6 weeks; then bi-weekly for the study duration. Semen was collected by eletroejaculation via rectal probe electrostimulation as, described [68]. Briefly, stimulation is provided by rhythmic pulsation from the stimulator, initially to 5 volts, with the voltage increasing over a 2 second period, with the stimulus maintained for 5–15 seconds and being reduced over 2 seconds. This rhythm is repeated with the maximum voltage increasing in 3-volt steps to a maximum of 20 volts. Stimulation can be repeated. Seminal emission is collected into a sterile container. Semen was centrifuged immediately after collection at 4°C and immediately frozen at −80°C. Semen cell pellets were washed 3× in cold PBS followed by the addition of 250 µl of *RNAlater* (Ambion, Austin, TX). Samples were immediately frozen and maintained at −80°C until use.

### SIV RNA isolation qRT-PCR and assay evaluation

Viral RNA levels were monitored in the seminal plasma and semen-associated cell fraction for 16 weeks after infection. Viral RNA was routinely isolated from 200 µL of cell-free, clarified peripheral blood or seminal plasma using the NucliSENS Isolation Kit (Biomerieux, Lyon, France) following the manufacturer's protocol.

The SIV RNA standard was transcribed from the pSP72 vector containing the first 731 bp of the SIVmac239-Gag gene using the Megascript T7 kit (Ambion Inc.). RNA was isolated by phenol-chloroform purification followed by ethanol precipitation. All purified RNA preparations were quantified by optical density. RNA quality was determined by Agilent bioanalyzer RNA chip (Agilent Inc., Santa Clara CA).

QRT-PCR was conducted in a 2-step process. First, RNA was reverse transcribed in parallel with an SIV-*gag* RNA standard using the gene-specific primer sGag-R 5′CACTAGGTGTCTCTGCACTATCTGTTTTG-3′under the following conditions: the 50 µL reactions containing 1× buffer (250 mM Tris-HCL pH 8.3, 375 mM KCl, 15 mM MgCl_2_), 0.25 µM primer, 0.5 mM dNTPs (Roche), 5 mM dTT, 500 U Superscript III RT (Invitrogen, Carlsbad, CA), 100 U RnaseOUT (Invitrogen), and 10 µL of sample. RT conditions were 1 hour at 50°C, 1 hour at 55°C and 15 minutes at 70°C. All samples were then treated with RNAse H (Stratagene) for 20 minutes at 37°C. All real-time PCR reactions used EZ RT PCR Core Reagents (Applied Biosystems, Foster City, CA) following the manufacturer's suggested instructions under the following conditions: the 50µL reactions containing 1× buffer (250 mM Bicine, 575 mM potassium acetate, 0.05 mM EDTA, 300 nM Passive Reference 1, 40% (w/v) glycerol, pH 8.2, .3 mM each of dATP, dCTP, dGTP, .6 mM dUTP, 3 mM Mn (OAc)_2_, .5 U uracil N-glycosylase, 5 U r*Tth* DNA Polymerase, .4 uM of each primer, and 10µL of sample template. Following 2 minutes at 50°C, the polymerase was activated for 10 minutes at 95°C, and then cycling proceeded at 15 seconds at 95°C and 1 minute at 60°C for fifty cycles. Primer sequences were adapted from those described by Cline et al [69], forward primer s-Gag-F: 5′-GTCTGCGTCATCTGGTGCATTC-3′, reverse primer s-Gag-R: 5′-CACTAGGTGTCTCTGCACTATCTGTTTTG-3′, and the probe s-Gag-P: 5′-CTTCCTCAGTGTGTTTCACTTTCTCTTCTGCG-3′, linked to Fam and BHQ (Invitrogen, Carlsbad, CA). All reactions were carried out on a 7300 ABI Real-Time PCR system (Applied Biosystems) in triplicate according to the manufacturer's protocols.

Preliminary experiments were done to evaluate the quantitation of blood, and seminal plasma viral RNA levels and their correlation with known viral RNA quantities spiked into blood or semen sampled from SIV-uninfected monkeys (data not shown). To determine the linear range of the assay, RNA extraction efficiency and potential inhibition of reverse transcriptase activity, known quantities of serially diluted SIVmac251 viral stock were spiked into samples of peripheral blood, or semen that were recovered from three SIV-naïve monkeys. No significant differences in extraction efficiency were observed between the blood and samples derived from other compartments (data not shown). Furthermore, using the extraction methods described above, we observed no inhibition of RT activity or diminution of input RNA as a consequence of RNAse activity present in any of the virus-spiked specimens (data not shown). These findings indicated, therefore, that it was appropriate to employ this technique to isolate viral RNA from study samples of peripheral blood and semen from infected rhesus monkeys. The analytical sensitivity of our quantitative RT-PCR assay was determined as described [44], [70].

### Detection of viral DNA

Cellular DNA was isolated using a QIAamp DNA Mini kit (QIAGEN). The number of SIV *gag* DNA copies was calculated as described previously [71], [72] and is shown as log-transformed copies per 1×10^5^ semen-associated cells. All target sequences were normalized to Albumen levels in genomic DNA in multiplex reaction performed in duplicate (Vic-labelled Taqman reagents, Applied Biosystems).

### Single genome sequencing of SIV envelope

Viral cDNA was diluted in 96-well plates to yield fewer than 30% wells positive for amplification to ensure that positive amplifications were a result of a single cDNA (Palmer et al., 2005). First-round PCR was carried out in a reaction mixture containing: 1× buffer (Platinum Taq HF Kit, Invitrogen), 0.2 mM dNTP mix, 2mM Mg_2_SO_4_, 0.2 µM primer OF6207 (5′ – GGGTAGTGGAGGTTCTGGAAG – 3′), 0.2 µM primer OR9608 (5′ – CTCATCTGATACATTTACGGGG – 3′), 0.025 units of Platinum Taq High Fidelity polymerase in a total volume of 20 µL. PCR mixtures were loaded into MicroAmp Optical 96-Well Reaction Plates (Applied Biosciences). PCR conditions were programmed as follows: 5 minutes at 94°C, 35 cycles of 15 seconds at 94°C, 30 seconds at 52°C, 4 minutes and 15 seconds at 68°C, followed by a final extension time of 10 minutes at 68°C [59]. For the second-round PCR, 2 µL of first-round PCR product was mixed with 1× buffer (Platinum Taq HF Kit, Invitrogen), 0.2 mM dNTP mix, 2mM Mg_2_SO_4_, 0.3 µM primer IF6428 (5′ – CGTGCTATAACACATGCTATTG – 3′), 0.3 µM primer IR9351 (5′ – CCCTACCAAGTCATCATCTTC – 3′), 0.025 units of Platinum Taq High Fidelity polymerase in a total volume of 20 µL. PCR conditions were: 5 minutes at 94°C, 45 cycles of 15 seconds at 94°C, 30 seconds at 51°C, 3 minutes and 30 seconds at 68°C, followed by a final extension time of 10 minutes at 68°C. Amplicons from cDNA dilutions resulting in less than 30% positive wells were sequenced at the Dana-Farber/Harvard Cancer Center DNA Resource Core.

Raw cDNA sequence data was assembled using GeneCodes Sequencher 4.8 DNA sequencing software. All assembled sequence contigs were manually corrected for individual ambiguous nucleotide errors and further quality controlled to exclude any amplicons derived from multiple templates according to that described by Learn et al, [73]. Nucleotide alignments were made using the GeneCutter algorithm as described below (http://www.hiv.lanl.gov/content/sequence/GENE_CUTTER/cutter.html).

### Analysis of SIV envelope diversity

The median and range of diversity was determined for each monkey, for both blood and semen, using a strategy suggested by Gilbert et al., that controls for the correlation of inter-sequence distances from the same animal and compared *env* divergence between animals receiving vaccine or sham-vaccinated controls. We modified the method described by Gilbert et al., to include a correction for small sample size bias (Giorgi and Bhattacharya, unpublished data), and restricted our analysis to animals those that had more than 3 sequences [74]. A nucleotide alignment of all sequences was generated with using GeneCutter, and then manually refined to maintain intact codons. The aligned sequences were trimmed to 2499 nucleotides, a region spanning the start codon of *env* through the first 17 nt of *nef*, to yield consistent phylogenetic signal among sequences, rather than variable signal due to 3′ gaps in some sequences but not others.

We used both Neighbor-Joining (NJ) and maximum likelihood (ML) for phylogenetic inference. The trees in figures 1,3,4,5, and 6 are from NJ via APE, version 2.5–3 [75] and BIONJ [76] with uncorrected pairwise distances and nodes with over 70% support from 1000 bootstrap replicates are labeled. The trees in the Supplementary data section were also obtained using Neighbor-Joining, APE and BIONJ with uncorrected pairwise distances and nodes with over 70% support from 100 bootstrap replicates are labeled. Use of an uncorrected substitution model is justified for representing within-host evolution because multiple mutations at the same site are unlikely to have occurred within 25% of the genome by 16 weeks post-infection. We inferred trees for the SM compartmentalization tests by PhyML [77] using the HKY85 substitution model, a discrete 4-parameter approximation to a gamma distribution of rate variation, and a term for invariant sites. Model parameters were also obtained via ML. We independently recomputed all trees in PAUP*, version 4.0b10 using ML substitution model parameters and NJ trees. Aside from minor differences in branch lengths and a tendency for NJ to yield negative lengths on some short branches, we did not find that results differed whether likelihood or distance-based tree optimality criteria were employed, regardless of the software implementation.

### Analysis of compartment-specific amino acid signatures

Signature detection utilized a phylogenetic correction to avoid over-counting amino acid substitutions among lineages of related sequences. To do this, we used methods described previously [78], and constructed a two-by-two contingency table for each amino-acid site in the alignment, populating the table with counts based on the immediate ancestor for each leaf in the tree. Columns in the contingency table correspond to whether or not the amino acid matches the ancestral state and rows correspond to whether the sequence is from blood or semen. Fisher's exact test identified significant deviations from equal proportions of amino-acid substitutions in blood versus semen-derived *env* sequences, with a false-discovery rate correction applied to the resulting p-values to accommodate multiple tests.

### Compartmentalization tests

To test for compartmentalization in contemporaneous blood- and semen-derived SIV *env* sequences, we used the Slatkin-Maddison test [52] as implemented in the HYPHY software package (v0.99b) as described by Pond et al. [53]. The SM test computes *s*, the observed number of migration events required to yield the observed distribution of blood and semen sequences on a tree. It then assesses significance by comparing *s* with a re-sampled null distribution, which is obtained by holding the tree topology constant and randomly permuting the order of blood and semen labels across all leaves on the tree, computing *s* for each permutation. Statistical support for compartmentalization is attained when a large proportion of permutations yield more migration events than for the empirical *s*. That is, if fewer than 5% of 10,000 replicate permutations have at most *s* migration events, then P<0.05.

### Viral recombination

Analysis for recombination was performed by the visual inspection of *Highlighter* plots and confirmed by using the G.A.R.D. analysis, where significance was assessed at (P<0.001) (13, 14).

### Methods for detecting positive selection

Protein-coding regions for all Env were tested for specific codons under positive selection using FEL. A Neighbor-Joining tree and nucleotide substitution model were computed for each aligned gene before FEL analysis [47]. Sites with greater nonsynonymous than synonymous substitution rates (dN>dS) and P<0.05 were taken as significant for positive selection. FEL results were not corrected for multiple testing, as according to Pond and Frost.

### Statistical inference and hypothesis testing

Statistical analysis was conducted using commercially available software GraphPad Prism version 4.0b (GraphPad Software, Inc. San Diego, CA 92130 USA). Linear regressions were conducted to determine the significance of correlations. Significance of differences between groups was determined using Kruskall–Wallis ANOVA as appropriate. Probability values of P<0.05 were interpreted as significant.

## Supporting Information

Table S1 Analysis of positively selected SIV env codons by fixed-effects likelihood test.
(0.03 MB DOC)

Text S1 Viral sequence diversity in the semen and blood after i.v. SIVmac251 infection. NJ and *Highlighter* analyses of sequences from control monkeys AX89, C171, DA2D, CK7J, CM8X (Fig. S1–S5), and vaccinated monkeys D135, AX93, AY47, AY89 and C179 (Fig. S6–S10). Shown on each tree are 2 weeks after infection (blood, purple circles semen, aqua circles) or post set point at 16 weeks after SIV challenge (blood, red circles or semen, blue circles) compared with inoculum sequences (black symbols). Brown points indicate sequences with three or more APOBEC-mediated G-to-A mutations compared with consensus sequences. Nucleotide polymorphisms in Highlighter plots are indicated as follows: A green, C cyan, G orange, T red, Other IUPAC code dark blue, Gap/no data grey.
(1.72 MB PDF)
