# Supplementary material for: Genital Tract Sequestration of SIV following Acute Infection
Source: PLoS Pathog. 2011 Feb 17;7(2):e1001293. doi: 10.1371/journal.ppat.1001293 (PMC3040679; doi:10.1371/journal.ppat.1001293)
Supplement: Table S1 — Analysis of positively selected SIV env codons by fixed-effects likelihood test. (0.03 MB DOC) [file ppat.1001293.s001.doc]

**Table S1:** **Analysis of positively selected SIV *env* codons by fixed-effects likelihood test.** Protein-coding regions for all Env were tested for specific codons under positive selection using FEL [47]. Sites with greater nonsynonymous than synonymous substitution rates (dN>dS) and p<0.05 were taken as significant for positive selection in vivo. Positively selected SIV *env* codons that overlap with other virus reading frames are shown in ***italicized boldface***.

| **ID** | **Codon** | **dN** | **dN-dS** | **p-value** |
| --- | --- | --- | --- | --- |
| AX93 | 146 | 19.75 | 258.7 | 0.0096 |
|  | 423 | 7.965 | 68.88 | 0.043 |
|  | 427 | 21.48 | 185.7 | 0.0041 |
|  | ***805*** | 42.30 | 365.8 | 0.012 |
| DA2D | ***759*** | 20.12 | 212.3 | 0.024 |
| AY47 | 427 | 58.06 | 1653.1 | 0.034 |
| AY89 | 373 | 17.58 | 166.7 | 0.032 |
|  | ***740*** | 11.60 | 110.0 | 0.032 |
|  | ***811*** | 11.05 | 104.8 | 0.034 |
| C171 | 99 | 19.44 | 247.8 | 0.0073 |
|  | 142 | 31.27 | 398.6 | 0.0014 |
| CM8X | 261 | 10.83 | 96.40 | 0.024 |
