# Supplementary figures and images for: Genital Tract Sequestration of SIV following Acute Infection
Source: PLoS Pathog. 2011 Feb 17;7(2):e1001293. doi: 10.1371/journal.ppat.1001293 (PMC3040679; doi:10.1371/journal.ppat.1001293)

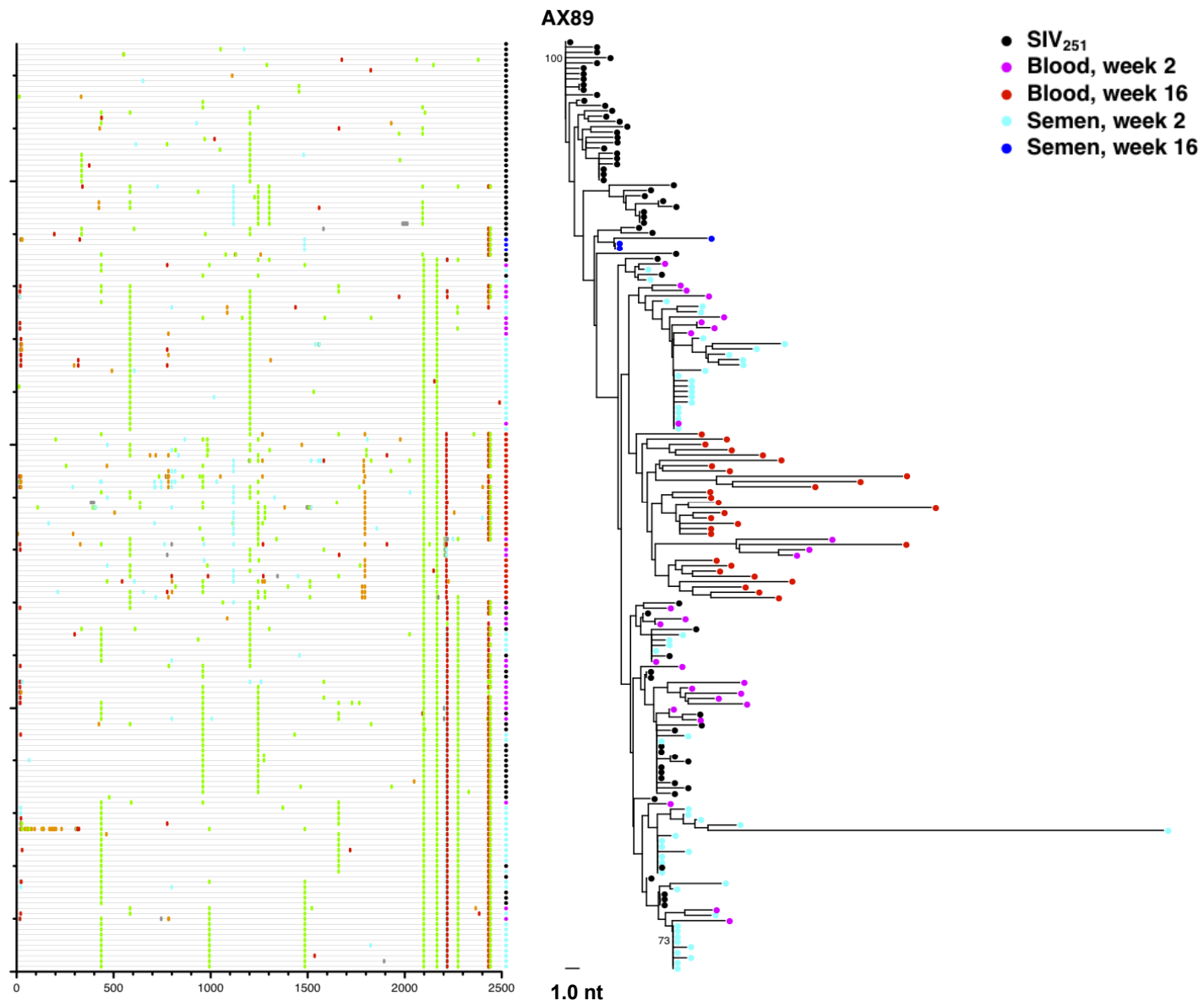

**Figure S1**

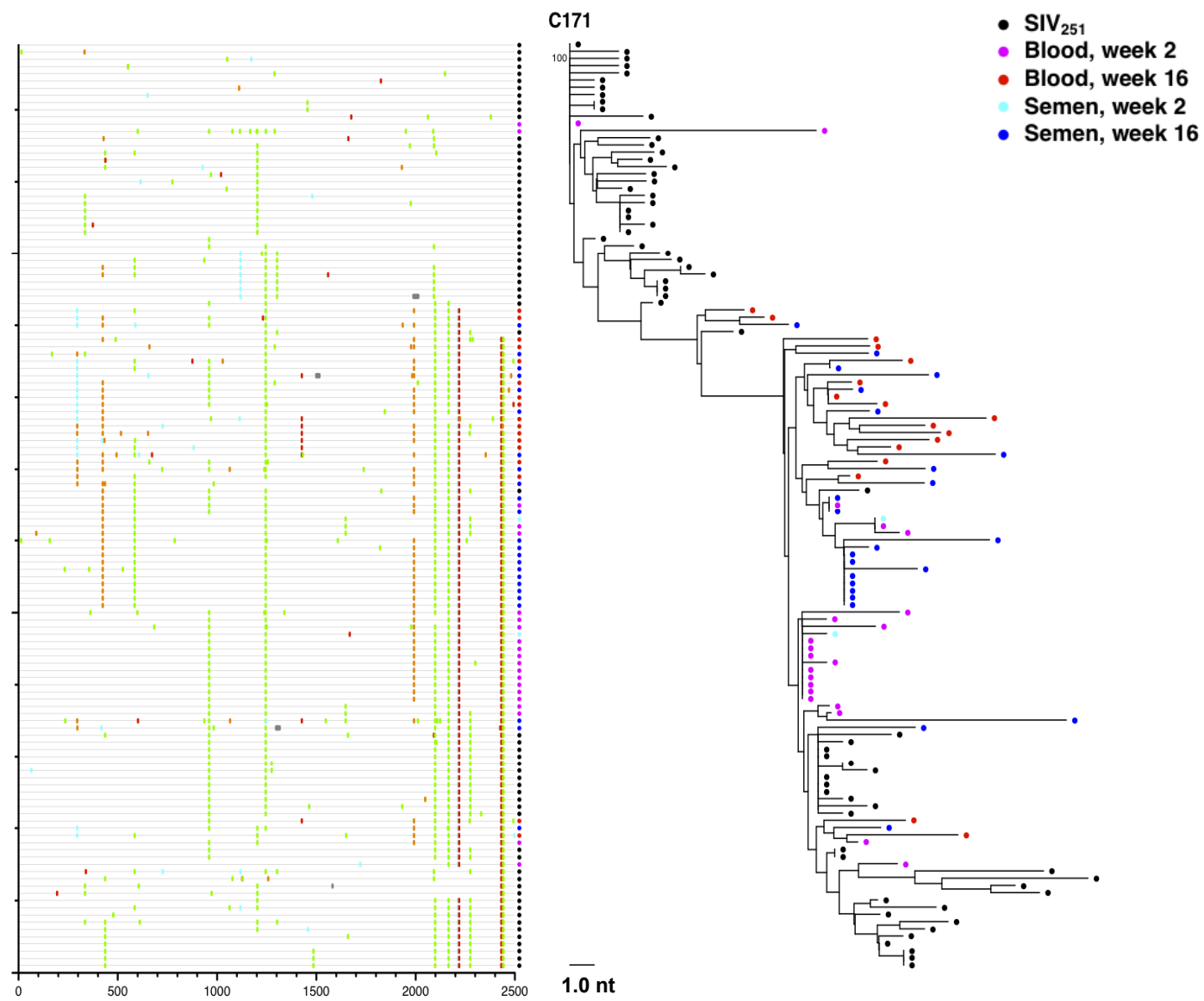

Figure S2

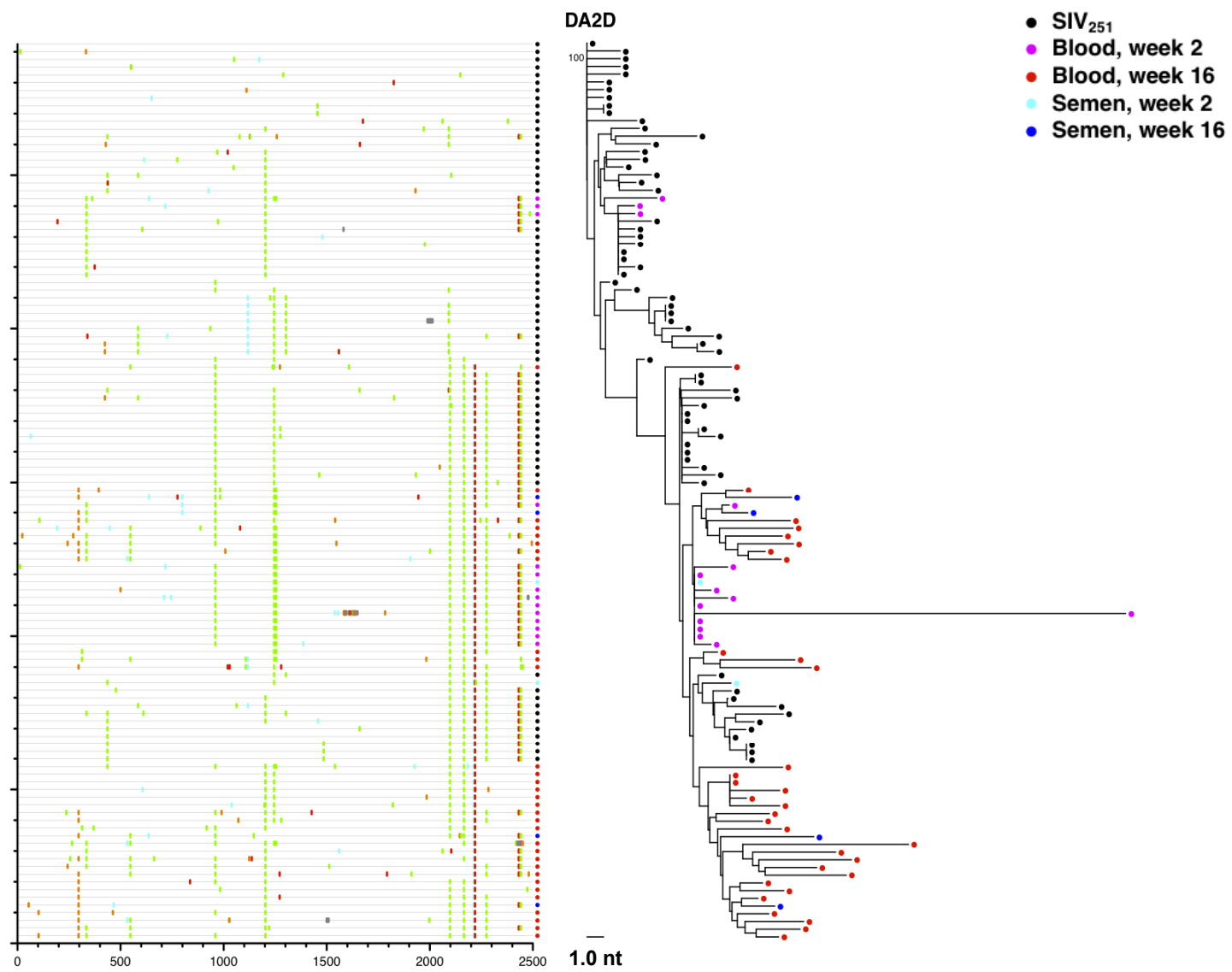

Figure S3

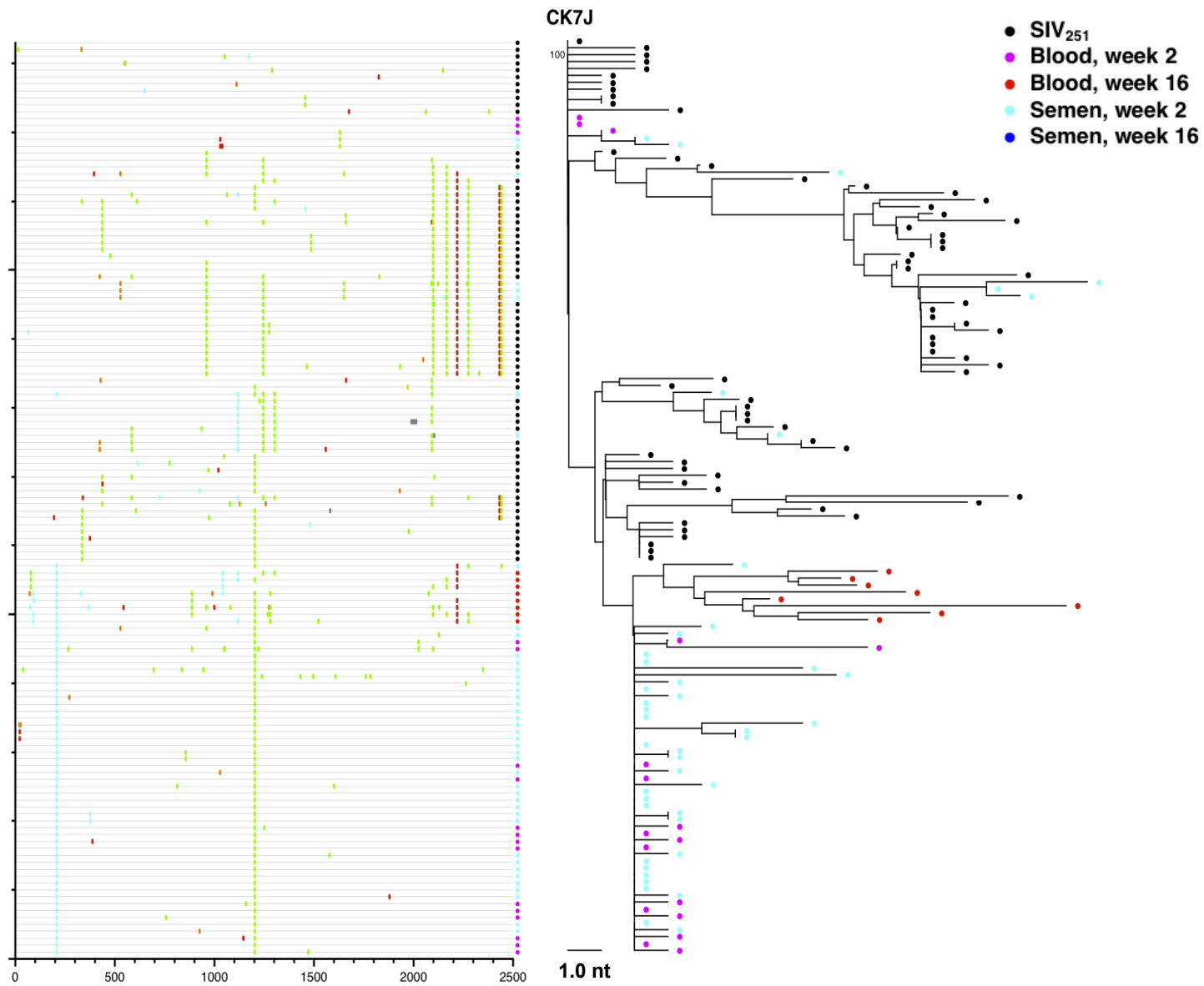

Figure S4

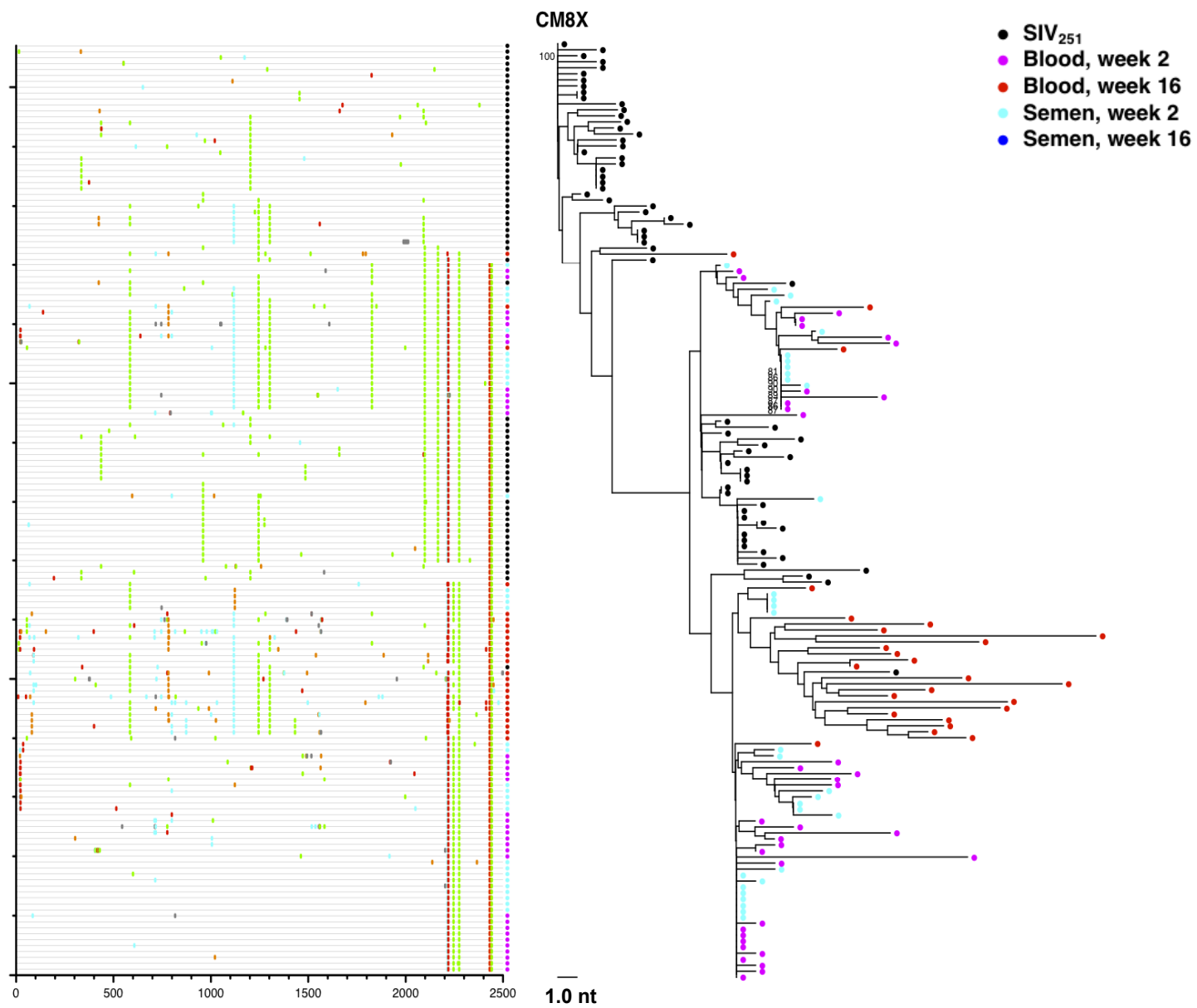

Figure S5

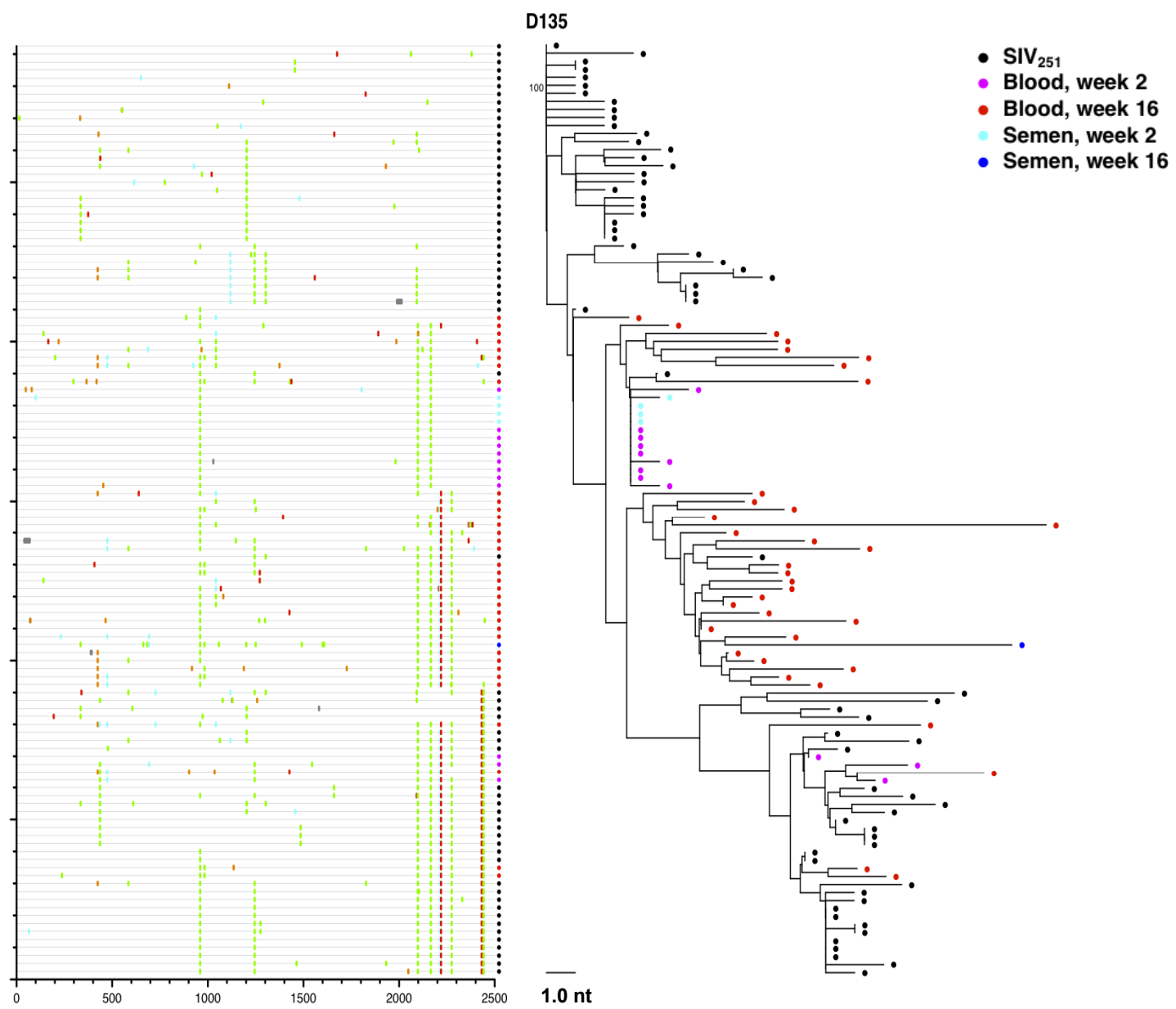

Figure S6

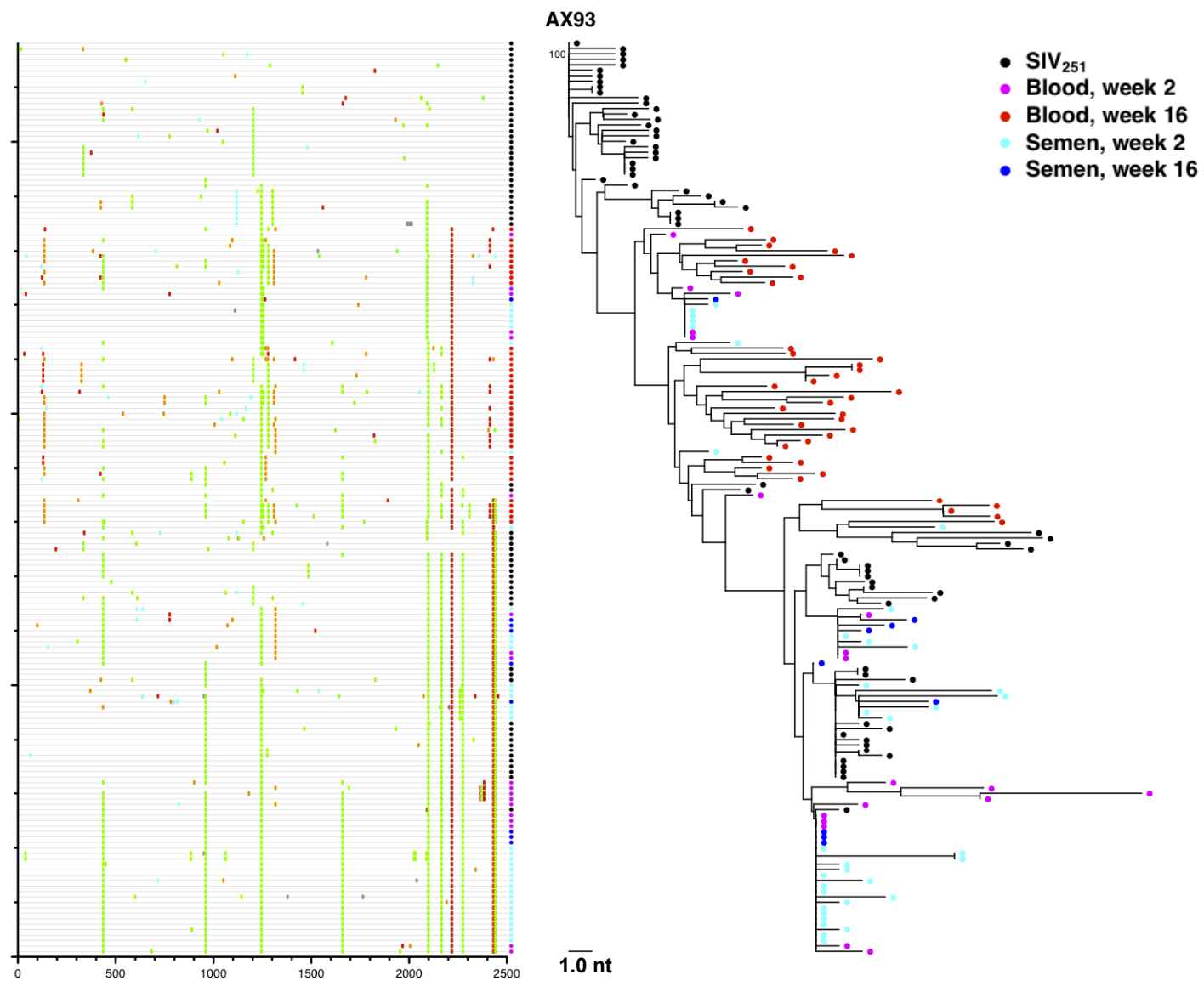

Figure S7

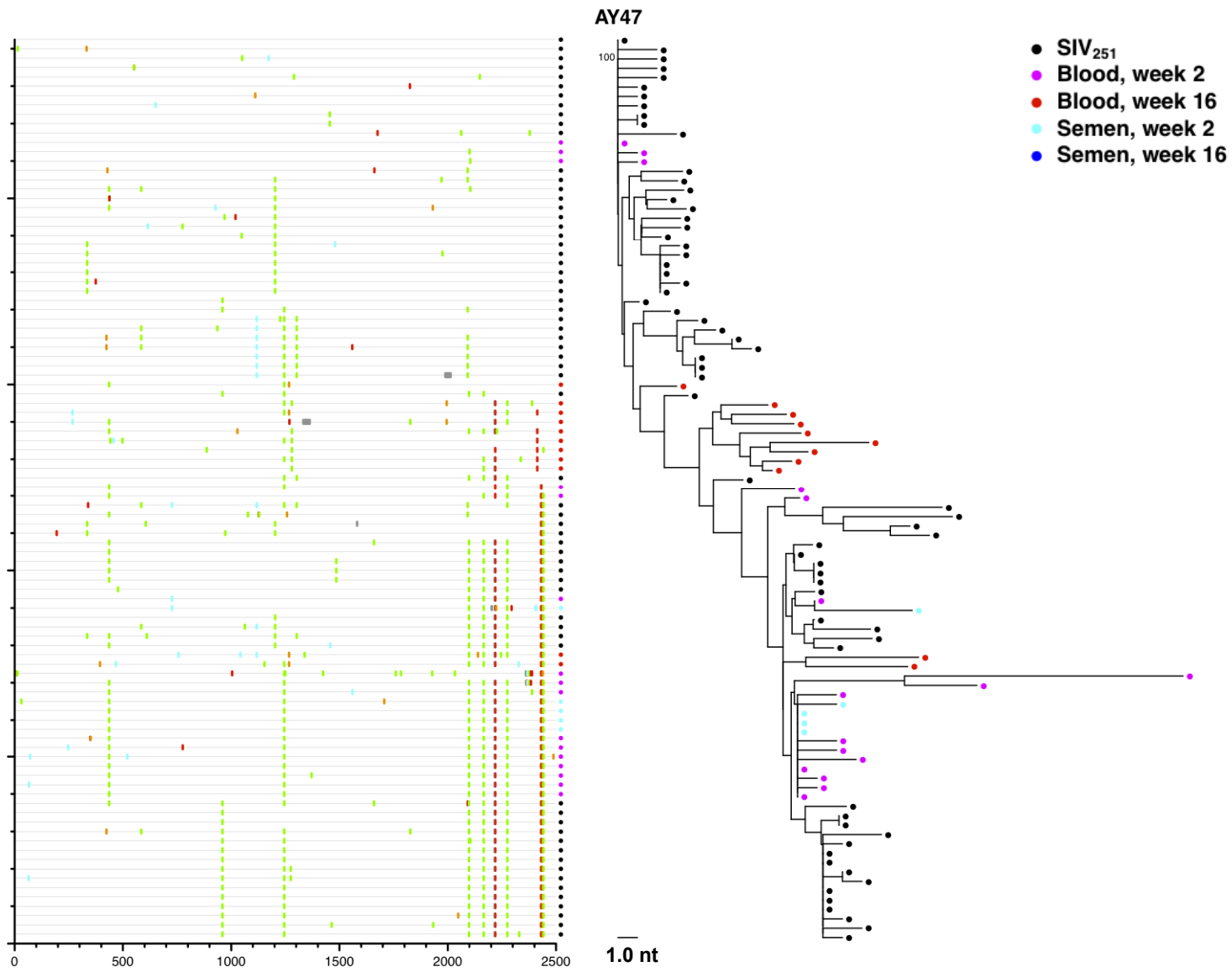

Figure S8

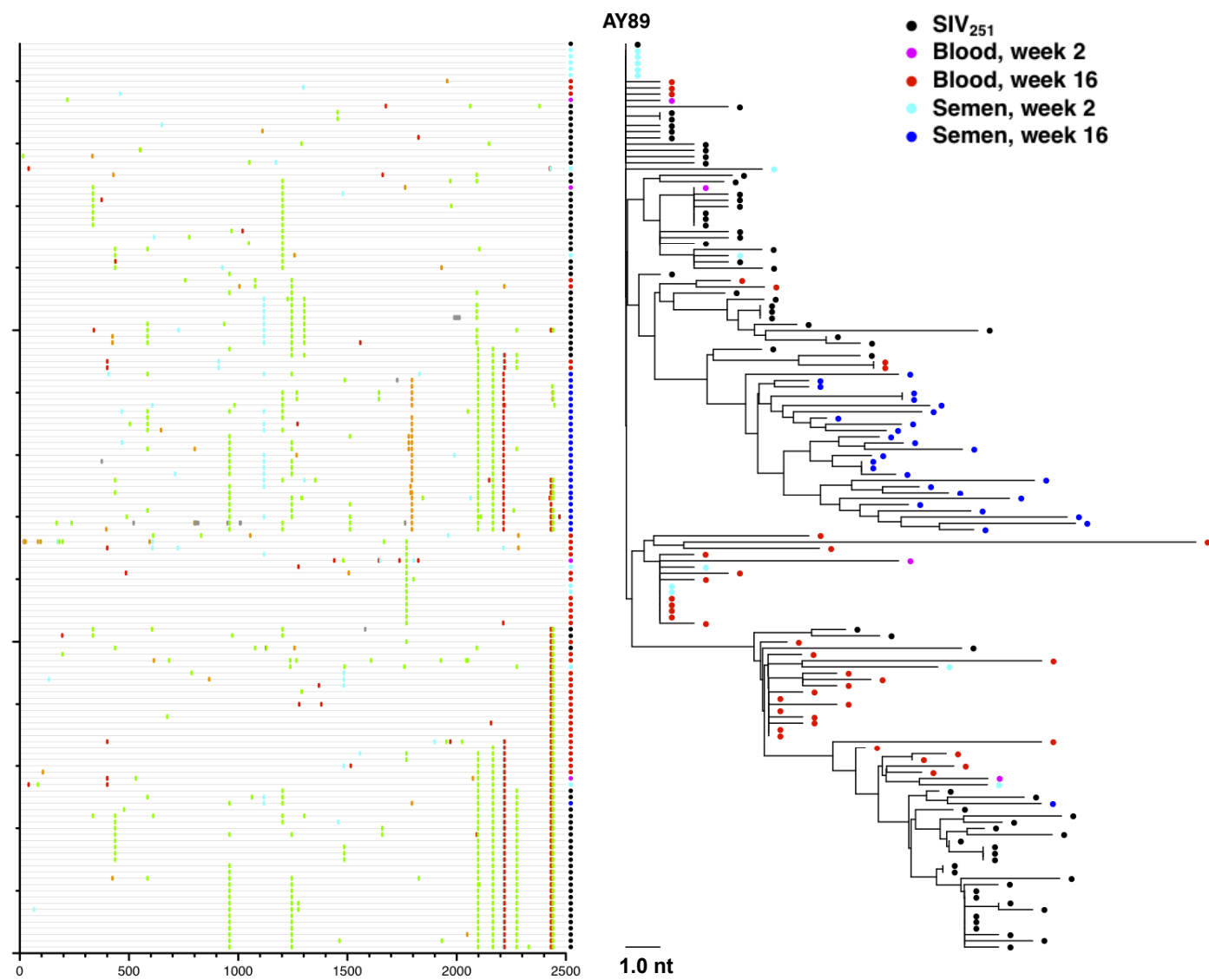

Figure S9

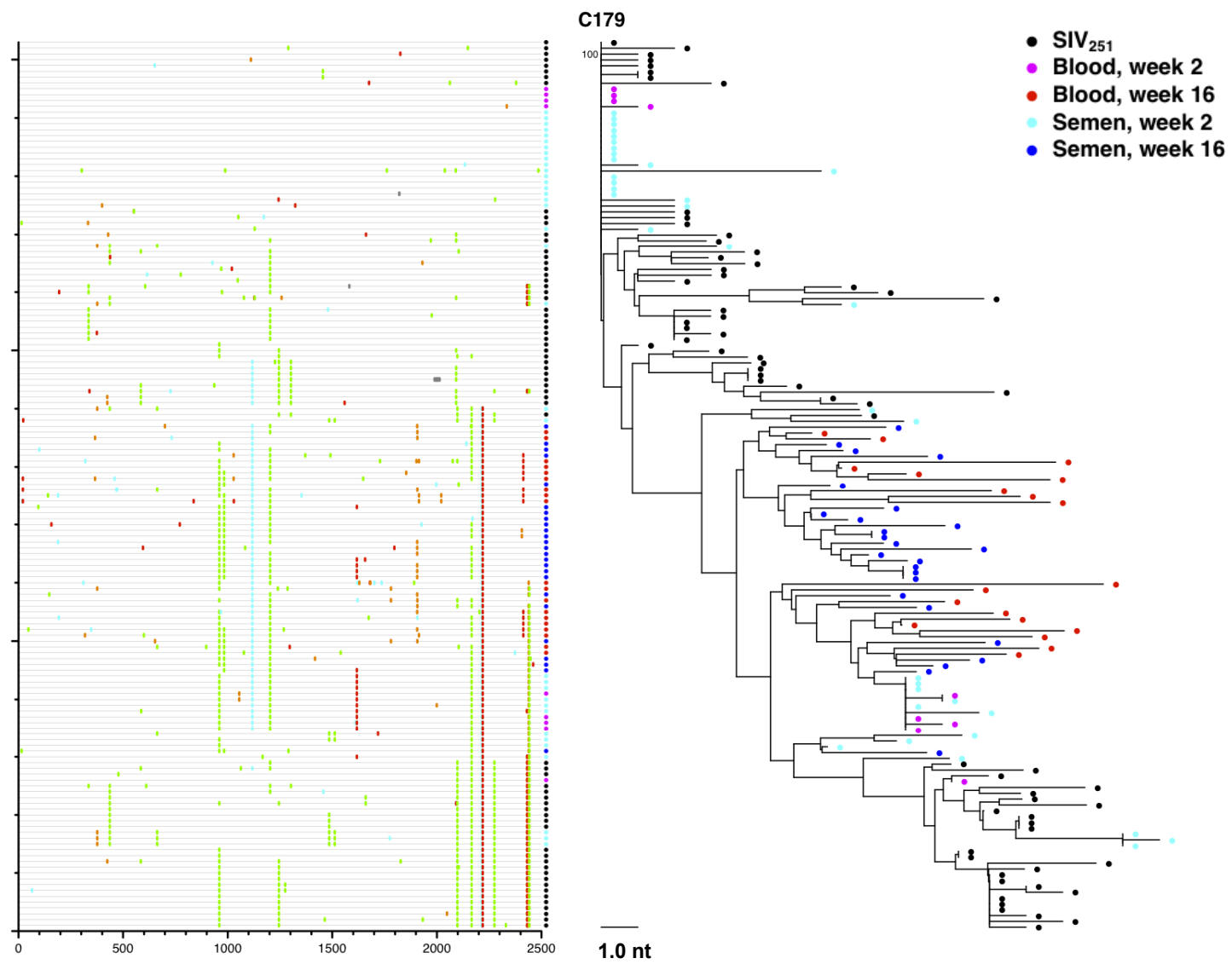

Figure S10

Supplement: Text S1 — Viral sequence diversity in the semen and blood after i.v. SIVmac251 infection. NJ and Highlighter analyses of sequences from control monkeys AX89, C171, DA2D, CK7J, CM8X (Fig. S1–S5), and vaccinated monkeys D135, AX93, AY47, AY89 and C179 (Fig. S6–S10). Shown on each tree are 2 weeks after infection (blood, purple circles semen, aqua circles) or post set point at 16 weeks after SIV challenge (blood, red circles or semen, blue circles) compared with inoculum sequences (black symbols). Brown points indicate sequences with three or more APOBEC-mediated G-to-A mutations compared with consensus sequences. Nucleotide polymorphisms in Highlighter plots are indicated as follows: A green, C cyan, G orange, T red, Other IUPAC code dark blue, Gap/no data grey. (1.72 MB PDF) [file ppat.1001293.s002.pdf]
